# Northward geographic diversification of a kleptoparasitic spider *Argyrodes lanyuensis* (Araneae, Theridiidae) from the Philippine Archipelago to Orchid Island

**DOI:** 10.1002/ece3.7910

**Published:** 2021-07-22

**Authors:** Mae Responte, Yi‐Fan Chiu, Po Peng, Rafe M. Brown, Chia‐Yen Dai, Yong‐Chao Su

**Affiliations:** ^1^ Graduate Institute of Medicine College of Medicine Kaohsiung Medical University Kaohsiung Taiwan; ^2^ Department of Biological Sciences and Environmental Studies College of Science and Mathematics University of the Philippines Mindanao Davao City Philippines; ^3^ Department of Biomedical Science and Environmental Biology College of Life Science Kaohsiung Medical University Kaohsiung Taiwan; ^4^ Biodiversity Institute Department of Ecology and Evolutionary Biology University of Kansas Lawrence Kansas USA; ^5^ Department of Medicine College of Medicine Kaohsiung Medical University Kaohsiung Taiwan

**Keywords:** Araneae, biogeography, distribution pattern, molecular phylogenetics

## Abstract

Oceanic islands are unique geographic systems that promote local adaptations and allopatric speciation in many of their highly endemic taxa. This is a common case in the Philippine Archipelago, where numerous unrelated taxa on islands have been inferred to have diversified in isolation. However, few cases have been reported in invertebrates especially among parasitic organisms. Here, we tested for biogeographical structure in novel populations of the “generalist" kleptoparasitic spider, *Argyrodes lanyuensis* Yoshida, Tso & Severinghaus, 1998 in the Philippines. Results showed that, in addition to Orchid/Lanyu Island, this species has a wide geographic distribution in the Philippine Archipelago. The estimated divergence time of this lineage using the mitochondrial cytochrome oxidase 1 (mt‐CO1) suggests that this species diverged *ca* 3.12 MYA, during the Pliocene. Two reciprocal monophyletic clades were elucidated in *A. lanyuensis*, but with limited differentiation across Pleistocene Aggregate Island Complex (PAIC) boundaries and modern‐day islands. However, in our analyses of morphological variation, we identified two phenotypically differentiated units in males (Orchid Island, Taiwan + Luzon, Philippine PAIC populations vs. Palawan + West Visayan + Mindanao PAIC populations). We infer that this species diverged in the southern portion of the Philippine Archipelago and only recently colonized Orchid Island. Our study provides new information on the extensive distribution of *A. lanyuensis* outside Orchid Island, Taiwan, but we documented a very limited geographically associated genetic variation. Our study points to behavioral phenomena such as foraging behavior as essential contributor to the evolutionary process of species diversification, in contrast to the traditionally invoked geographic drivers of divergence.

## Graphical abstract

The kleptoparasitic spider, *Argyrodes lanyuensis* Yoshida, Tso & Severinghaus, 1998, has been considered endemic in Orchid/Lanyu Island, Taiwan, since it was first described. However, our study revealed novel populations of *A. lanyuensis* on at least six islands of the Philippine archipelago. We inferred the diversification and colonization patterns of this species and assessed whether a strongly subdivided geographic system was effective in generating a pronounced geographically structured variations among its populations. It was revealed that this species diverged from the southern portion of the Philippine Archipelago and only recently colonized Orchid Island.

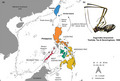

## 1 INTRODUCTION

Oceanic island chains usually host high levels of endemic terrestrial biodiversity because of strong geographic isolation, which promotes the partitioning of their fauna and flora (Gillespie, 2007; Lomolino et al., 2010). Dispersal plays an important mechanism in the process of diversification of taxa in an oceanic island (De Queiroz, 2005; Gillespie et al., 2012). Once a historical oceanic island emerged above the surface of the ocean, it is then available for colonization of taxa from distant land areas (Cowie & Holland, 2006). However, effective colonization to oceanic islands particularly by terrestrial biota depends on several factors, such as climatic conditions, wind speed variation, local adaptation, sizes of islands, distance to source biota, and geographic boundary fluctuations. (Leihy & Chown, 2020; Lomolino et al., 2010; Whittaker et al., 2007). All of which may contribute to the historical subdivision of populations on oceanic islands.

The Philippine Archipelago is known as one such highly partitioned case: a dynamic, highly fragmented geographical template. It consists of more than 7,000 oceanic islands situated at a unique location—spanning portions of the Australasian and Asian faunal regions (Brown & Diesmos, 2009; Brown et al., 2013; Lohman et al., 2011). It hosts substantial genetic structure, both within species and among highly differentiated lineages (Brown et al., 2016; Hosner et al., 2014; Siler, Oaks, et al., 2012; Su et al., 2014; Wood et al., 2020). The subdivision of populations, species, and even higher taxa have been hypothesized to be the result of dynamics current and historical geographic processes of the archipelago (Hall, 1998, 2002; Yumul et al., 2009). With the relatively clear understanding of the geographic boundaries, and dynamic nature of their corresponding geological history, reassessment of species diversity and mechanisms of diversification has been explored comprehensively in multiple clades (Hosner et al., 2014; Linkem et al., 2010; Siler, Jones, et al., 2012; Weinell & Brown, 2017). This resulted in the identification of localized evolutionary trends and many instances of allopatric speciation following bouts of dispersal (Barley et al., 2020; Brown et al., 2016; Oaks et al., 2019; Siler et al., 2010). It is intuitive to consider that pronounced subdivision of the Philippine Islands might cause or be related to diversification, presumably resulting in the formation of new endemic species once their ancestors invaded relatively isolated islands (Heaney, 1985; Inger, 1954). However, whether such species continued to expand their range via recent dispersals among islands has rarely been reported (but see Brown et al., 2010; Siler et al., 2014).

**FIGURE 1:**
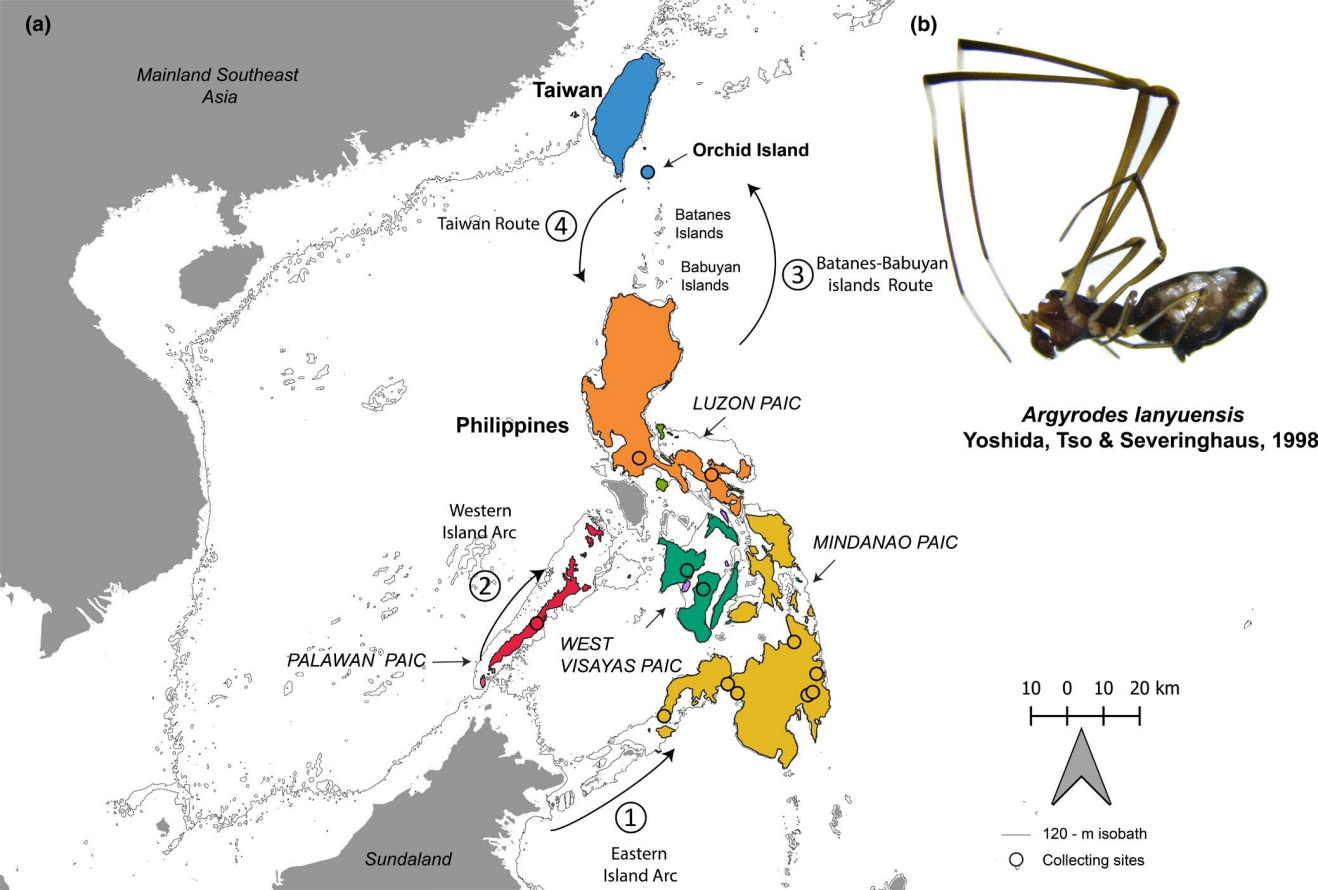
Paleogeological features of Taiwan and the Philippine Archipelago (Hall, 1998). (a) Arrangement of Taiwan—Philippine archipelago during the Pleistocene epoch, <2 Million Years ago (MYA) with the −120 m contours (gray) of the Pleistocene aggregate island complexes (PAICs). Taiwan was connected with mainland China, while Philippines formed different PAICs as indicated by each color. We hypothesized south‐to‐north colonization via **①** eastern island arc hypothesis, **②** western island arc hypothesis, and **③** Batanes‐Babuyan islands route; and north‐to‐south colonization via **④** Taiwan route. (b) Photograph of male *Argyrodes lanyuensis* Yoshida et al., 1998

The Philippines is located approximately 390 km south of Taiwan, but Orchid/Lanyu Island (Taiwan) and the Batanes and Babuyan island groups (Philippines) span the intervening seas with a series of small island chains (Figure 1a). Initially documented on Orchid/Lanyu Island, the kleptoparasitic spider, *Argyrodes lanyuensis* (Figure 1b), has been considered endemic to this small island since it was described in 1998 (Yoshida et al., 1998). However, our recent sampling of argyrodinae spiders in the Philippines has revealed the occurrence of *A. lanyuensis* in at least six of the archipelago's islands (Figure 1a). We used this species to assess whether a strongly subdivided geographic system (the oceanic portion of the archipelago) would be effective in generating pronounced geographical structure in genetic variation among populations of this kleptoparasitic spider. The foraging behavior of the subfamily Argyrodinae is remarkable in that they rely on either araneophagy or kleptoparasitism—or sometimes both—as their main feeding strategy (Cobbold & Su, 2010; Vollrath, 1979; Whitehouse, 2011). *Argyrodes lanyuensis* is closely related to the Philippine endemic *A. tripunctatus* Simon 1877, and two Australasian species, *A. nasutus* Pickard‐Cambridge 1880, and *A. rainbow* Roewer 1942 (Su & Smith, 2014). Based on the first reports of Yoshida et al. (1998), *A. lanyuensis* forages prey items and silk from the webs of a wide range of orb‐weaving spider hosts, that is, *Nephila*, *Gasteracantha,* and *Cyrtophora*. Aside from orb‐weaving hosts, it was also observed to hunt prey items and consume silk on *Achaearanea* (Theridiidae) host. Thus, an ecological “generalist” kleptoparasites like *A. lanyuensis* tend to have high tolerance on a wide array of spider hosts than ecological “specialist” kleptoparasites which specifically utilize one species/genus of orb‐weaving hosts (e.g., *A. fissifrons* and *A. miniaceus* kleptoparasites). Since most of the geographic variability, biogeography, and individual species distributions of Argyrodinae on oceanic islands have not been fully characterized, we focused on *A. lanyuensis* as a fitting representative of ecological “generalist” kleptoparasitic spiders to distinguish it from “specialist” kleptoparasites.

The Pleistocene Aggregate Island Complex (PAIC) model of speciation (Inger, 1954; Heaney, 1985, 1986; review: Brown & Diesmos, 2002, 2009) has been used as an operational hypothesis to generate testable predictions related to the analysis of diversification patterns among Philippine biota (Evans et al., 2003; Sánchez‐González & Moyle, 2011; Su et al., 2014). The Pleistocene glacial cycles (between 2.5 MYA to 18 KYA) resulted in the repeated rising and lowering of sea levels (100–140 m). In the Philippines, this led to the repetitive isolation and formation of land bridges between neighboring islands separated by shallow seas (Figure 1a). With the tracing of bathymetric contours (100–140 m) within this period, Pleistocene islands can be estimated with the maximum extent of land bridges. This resulted in six major larger island‐amalgamations known as PAICs: Luzon, Mindanao, Western Visayas, Mindoro, Sulu, and Palawan (Brown & Diesmos, 2002; Heaney, 1985, 1986). These paleoisland connections among islands in the Philippines served as a basis for predicting patterns of species diversity and distribution. To date, several vertebrate taxa like mammals, lizards, frogs, and birdsshowed nearly complete concordance to PAIC boundaries (Evans et al., 2003; Heaney, 1985, 1986; McGuire & Alcala, 2000; Sánchez‐González & Moyle, 2011). However, applying the PAIC speciation model to highly dispersive arthropod species is sparse in literature, except for one pilot study (Su et al., 2014). Even though it has not been utilized more often to terrestrial invertebrate species due to the characteristic of flight and ballooning, it is also worth noting that this speciation model has been used to explain the diversification patterns of widely distributed volant mammals and birds (Heaney et al., 2005; Sánchez‐González & Moyle, 2011).

The predictions derived from a strict interpretation of the PAIC Paradigm would include (1) a homogenized (or nearly so) gene pool of island populations within PAICs and (2) limited gene flow, leading to pronounced geographical structure, among and between PAICs. It follows, then, that if a particular taxon colonized the archipelago before or during the Pleistocene, the distribution of its species (or populations) would likely be found today in concordance with the PAIC model's six major faunal regions. The Philippines Archipelago has a dynamic geologic history (Hall, 2002; Yumul et al., 2003, 2009), which likely influenced the diversification of its fauna and flora (Brown & Diesmos, 2009; Brown et al., 2013). Therefore, we assumed that heterogeneous, interrupted, and partitioned geographic template of land area throughout the archipelago might have led to distinct populations of *A. lanyuensis* across oceanic islands including the island banks stretching north toward Taiwan and Orchid Island (Figure 1c). However, if we consider the dispersal ability of spiders through long‐distance ballooning (Bell et al., 2005; Bishop & Riechert, 1990), then we would expect to see little to no differentiation of *A. lanyuensis* populations, as it would greatly affect the gene flow of this species. Additionally, the behavior of this species, which is a generalist kleptoparasite, would also explain a little to no differentiation of taxa because generalists do not need to specifically adapt to a particular host (Su et al., 2018).

To ascertain how *A. lanyuensis* may have dispersed and colonized in the Philippine Archipelago and Orchid Island, we first update its geographical distribution and used time‐calibrated phylogenetic analyses. We infer the ancestral area range evolution using biogeographical reconstruction models. Initially, we hypothesized that this species diverged from Sundaland and colonized Philippine islands via Eastern and Western arcs (Figure 1a; Route 1, 2), through the northern‐most islands (Babuyan and Batanes island groups; Route 3), and eventually colonized Orchid Island, as suggested by the results of Su and Smith (2014). Alternatively, if the current distribution of the species came about by recent southward colonization (<1MYA), the species may have originated on Orchid Island (Route 4; Figure 1a) and subsequently colonized the Philippines via the Taiwan‐Batanes‐Babuyan island chain (Dickerson, 1928; Esselstyn & Oliveros, 2010; Oliveros et al., 2011). Thus, we undertook the current study to test the north‐to‐south versus south‐to‐north predictions derived from PAIC and analyze dispersal or vicariance events.

## 2 MATERIALS AND METHODS

### 2.1 Taxon sampling

We collected *A. lanyuensis* samples from the main islands in the Philippines and Taiwan between 2005 and 2007, and from July to August 2019. We found *A. lanyuensis* at only 13 collection sites on six islands: Orchid Island, Luzon, Palawan, Negros, Panay, and Mindanao (Figure 1a). Samples were collected from the webs of orb‐spinning spiders of families Araneidae, Tetragnathidae, and Uloboridae. We preserved the specimens in 95% ethanol and stored at −30°C, for subsequent morphological examination and DNA extraction. All specimens were deposited in Evolution and Ecological Genomics (EEG) Laboratory, Kaohsiung Medical University, Kaohsiung, Taiwan. Specific collection information and sample accession number for each specimen are reported in Appendix 1.

### 2.2 Morphological variation

To assess the geographic variation of *A. lanyuensis* populations from the Philippines and Orchid Island, we examined adult specimens for variation in continuous morphometric measurements. Male (*n* = 37) and female (*n* = 38) samples were observed under a Leica stereomicroscope. We embedded each specimen in a gel‐loaded calibration slide (1 division = 0.1 mm; 1 division = 0.01) and used tethered Nikon camera D5600 to capture high‐resolution images (Appendix 5). We utilized *measure3* software (Tsai, 2021; https://github.com/yucenwan/Spider‐measure) to generate calibrated measurements of specific body characteristics from captured high‐resolution images. We normalized body morphometrics using carapace length, following character definitions of Yoshida et al. (1998). The measured body characteristics include total length (TL), carapace length (CL), carapace width (CW), total length of each leg (L1TOT; L2TOT; L3TOT, and L4TOT)), and the length of each leg (I‐IV) segment: femur (L1F‐L4F); tibia + patella (L1PT‐L4PT); metatarsus (L1M‐L4M), and tarsus (L1T‐L4T). We additionally measured palp morphometrics from the male specimens, which include total palp length (PL), bulb length (BL), median apophysis (MA), accessorial apophysis (AP), and embolus length (EL). Bulb length was used to normalize all the palp morphometrics. All body and palp measurements used in this study were displayed in Appendix 5. Variation in morphometric dimensions (separately for males vs. females) was summarized in Principal Component Analysis (PCA) using the “prcomp” function in R 3.6.1 (R Core Team, 2017). Data visualization was carried out using the R package *ggfortify* (Horikoshi & Tang, 2018). We used nonlinear iterative partial least squares (NIPALS, followed Wold, 1973) in which the algorithm conducts local regressions using the latent components to predict and impute missing values caused by poor preservation conditions (Female, *n* = 9; 1.03% of the data matrix; Male, *n* = 50; 4.83% of the data matrix). To avoid multicollinearity problems among the measurements of our morphological data, we followed Vignon (2011) to adopt the Partial Least Square–Discriminant Analysis (PLS‐DA), assessing if individuals clustered into geographical distributions based on morphology. We used the “plsda” function within the R package *mixOmics* (Rohart et al., 2017), where all measurements were included as response variables. Permutational test with 9,999 repetitions was performed based on cross‐model validation procedures, where estimation of the classification error rate (CER) was used as the test statistics. Additionally, the function “pairwise.MVA.test” in the same R package was implemented for pairwise comparisons of clusters.

### 2.3 DNA extraction, marker choice, and PCR amplification

We extracted the genomic DNA from legs and prosomal tissues of preadult and adult specimens following the Maxwell® RSC Blood DNA Kit AS1400 protocol. Tissues were homogenized in 300 μl Lysis Buffer and 30 μl Proteinase K (PK) Solution and incubated at 56°C for 2 hr. We purified the genomic DNA through the Maxwell^®^ RSC Instrument following the manufacturer's instructions. The extracted genomic DNA was stored at −30°C condition until used for polymerase chain reaction (PCR) amplification.

We sequenced the mitochondrial cytochrome oxidase I (CO1) partial gene region, which is an effective genetic marker in species identification and taxonomic delimitation (Hebert et al., 2004), especially for invertebrates (Cao et al., 2016; Carew et al., 2007; Gutiérrez et al., 2014). The CO1 fragment was targeted and amplified using primer pairs, *CO1‐F* and *CO1‐r* designed by Su and Smith (2014). PCR amplification was performed in a TurboCycler 2 thermal cycler (TCST‐9622, Taiwan) with a total volume of 25 μl with 12 μl of premix, 10 μl of nuclease‐free water, and 0.5 μl to each of the primers. PCR products were visualized through 1.5% agarose gel electrophoresis to check amplified DNA fragments of the expected size and sequenced at the genetic sequencing facility of Genomics Co. Ltd., Taiwan.

### 2.4 Sequence alignment and molecular data analysis

We filtered all the sequences according to the quality control reports and obtained a total of 95 CO1 sequences. Some samples used in morphological analyses have poor quality and thus were not included in the population genetic analyses. Contigs were generated from merged forward, and reverse, sequences and their consensus sequences were aligned using Genious Prime 2020.2. Alignment was refined manually to generate a complete alignment of 840 base pairs.

We reconstructed a time‐calibrated phylogenetic tree using BEAST v1.10.4 (Drummond et al., 2012). We incorporated seven species (nine sequences in total) from GenBank as an outgroup (Appendix 1b). Species included in the outgroup are the closest relatives of *A. lanyuensis* according to the phylogenetic tree inferences of Su and Smith (2014). The program jModelTest2 v. 2.1.10 was used to calculate the best‐fit nucleotide substitution model for the CO1 gene using the Akaike Information Criterion (AIC) (Posada, 2008). The GTR+I + G best‐fit nucleotide substitution model, Yule process speciation tree model prior (Heled & Drummond, 2012), and the uncorrelated lognormal relaxed clock model (Drummond et al., 2006) were applied for node age time calibration. We used the ucld.mean = 0.0112 site^−1^ My^−1^ based on the spider mitochondrial substitution rate estimates (Bidegaray‐Batista & Arnedo, 2011; Kuntner et al., 2013) with an arbitrary standard deviation (ucld.stdv = 0.01). The MCMC parameters were fixed to 1 × 10^9^ generations with tree sampling every 1 × 10^4^ generations, after conducting preliminary runs (chain length 1 × 10^8^ and 5 × 10^8^). Tracer v.1.7.1 was used to determine burn‐in (discarded the first 10% of the trees) and to check the effective sample sizes (ESS ≥ 200; Rambaut et al., 2018). Maximum clade credibility (MCC) tree was then generated using the program TreeAnnotator v.1.8.4 (Rambaut & Drummond, 2010) and visualized using FigTree v.1.4.3 (Rambaut, 2014).

Additionally, nucleotide and haplotype diversity of the in‐group sequences were calculated based on the PAIC boundaries and current island boundaries using DnaSp v.6.12.03 (Rozas et al., 2017). Haplotype networks were also created in TCS v.1.21 (Clement et al., 2000) and displayed as a final network using tcsBU v.1.0 (Múrias dos Santos et al., 2016). We conducted an isolation by distance (IBD) test among PAIC islands through Mantel's test of correlation between Edward's distances and Euclidian geographic distances. IBD test was implemented in R package “adegenet” using the *mantel.randtest* function (Jombart, 2008). Cline and distant patches of points were checked using the 2‐dimensional kernel density estimation (kde2d) in R package “MASS.” Gene flow among current islands was further assessed by calculating pairwise Fixation indices (*F*_ST_) using the R package “StAMPP” (Pembleton & Pembleton, 2013).

### 2.5 Biogeographical analyses

The ancestral geographic ranges were reconstructed by two programs: R package “BioGeoBEARS” (Matzke, 2014), and Reconstruct Ancestral State in Phylogenies (RASP) (Yu et al., 2015). The best‐fit historical biogeographical model selection was conducted among six available models in “BioGeoBEARS”: DEC, DEC+j, DIVALIKE, DIVALIKE+j, BAYAREALIKE+j (Matzke, 2014). We applied the best‐fit historical model (BAYAREALIKE+j) with the highest corrected Akaike information criterion (AICc) weights to the time‐calibrated BEAST trees dataset and consensus tree dataset. Additionally, we applied the Bayesian Binary MCMC (BBM) and Statistical Dispersal‐Vicariance models in RASP as alternative biogeographical reconstruction analyses.

We designated the geographical distributions of *A. lanyuensis* according to PAIC islands, while the known geographical distribution of the outgroup was based on the descriptions from World Spider Catalog (2020) and other published literature. There were five current distinct geographical areas included for the in‐group: Orchid Island (A), Luzon PAIC (B), Palawan PAIC (C), West Visayas PAIC (D), and Mindanao PAIC (E). Five geographical areas were also included for the outgroup, namely Sundaland (F), Papua New Guinea (G), Japan (H), China (I), and Australia (J). The geographical range allowed at each node was set up to four geographical areas since no extant species occupied more than four geographical areas. Additionally, the density of evolutionary events such as dispersal and vicariance events was calculated and visualized along a time‐calibrated tree.

## 3 RESULTS

*Argyrodes lanyuensis* samples were collected from 13 sampling sites distributed across Orchid Island, Taiwan, the main (northern) component of Luzon Island, its southern Bicol Peninsula, Palawan, Negros, and Panay islands; plus, the northern, eastern, and southwestern (Zamboanga Peninsula) faunal subregions of Mindanao Island. Our sampling efforts have also reached the Ryukyu Islands (Japan) and Green Island (Taiwan). Additionally, we surveyed Cebu, Samar, Leyte, and Mindoro (Philippines), but did not find *A. lanyuensis* on these islands (2005 to 2019). At present, *A. lanyuensis* has a geographical distribution including the Philippine faunal regions of the Luzon, Palawan, West Visayas, and Mindanao PAICs, in addition to the original records (Yoshida et al., 1998) from Orchid Island, Taiwan.

**FIGURE 2:**
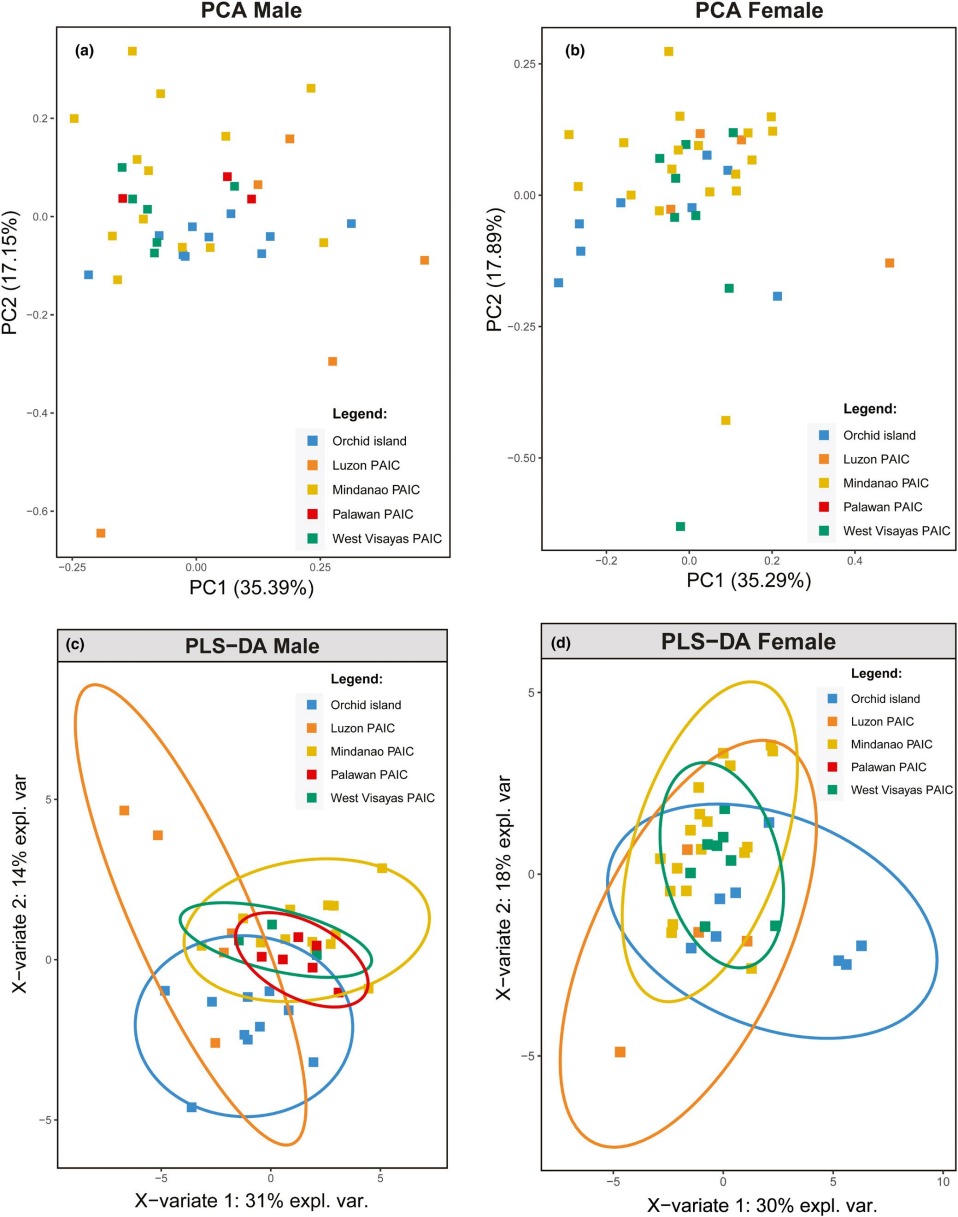
Principal component analysis (PCA) and partial least square‐discriminant analysis (PLS‐DA) score plots of male (a–c) and female (b–d) *A. lanyuensis* based on 28 and 23 morphometrics, respectively. Individuals are plotted against components 1 and 2 with 95% confidence ellipse for PLS‐DA plot. The lists of the characters used in these analyses are in Appendices 3 and 5

We analyzed the measurements of morphological characters of *A. lanyuensis* males (*n* = 37) and females (*n* = 38) using PCA with 28 and 23 variables, respectively (Appendix 3). We then classified and sorted samples into Mindanao, the West Visayas, the Palawan, the Luzon PAIC, and the Orchid Island. Palawan female samples were not included because adult specimens were not available. The PCA showed limited clustering to both *A. lanyuensis* males and females across different geographic areas (Figure 2a,b; Appendix 4). Although, we observed three samples from Luzon PAIC that deviated from the main male clusters (Figure 2a). The first principle component (PC) accounted for 35.39% of the variance, and the second PC explained 17.15% of the variance for male morphometrics. The first PC explained 35.29% of the variance in females, and the second PC accounted for an additional 17.89%. Overall, we observed no PAIC‐based clustering or divisions in males and females in the PCA results.

Alternatively, we used the PLS‐DA, which emphasized a dimension reduction technique for handling multicollinearity data (Vignon, 2011), to detect the morphological clustering among samples. Because individual samples were assigned according to PAICs a priori, the PLS‐DA score plot was able to discriminate PAIC clusters in both males and females (Figure 2c,d). For males, we identified one cluster (Orchid Island) that was clearly separated from the other samples, while Mindanao PAIC, Palawan PAIC, and West Visayas PAIC samples merge into a single overlapping cluster (Figure 2c). The Orchid Island cluster was significantly different with Mindanao PAIC cluster (Orchid Island vs. Mindanao PAIC: CER = 0.23741, *p*‐value < .05; Table 1a) and Visayas PAIC cluster (Orchid Island vs. West Visayas PAIC cluster: CER = 0.191, *p*‐value < .05; Table 1a). The Luzon PAIC samples were scattered with one sample overlapped with Orchid Island cluster and two samples overlapped with the rest of the PAIC samples. This cluster was significantly different with Mindanao PAIC cluster (Luzon PAIC vs. Mindanao PAIC: CER = 0.111, *p‐*value < .05; Table 1a). The overall discrimination method based on PLS‐DA among the male samples was found to be significant (CER = 0.512, *p‐*value < .05; Table 1a). Thus, we inferred two morphologically discrete clusters for male data as Mindanao+Palawan+West Visayan populations and Luzon+Orchid Island populations were undifferentiated (Figure 2c). In contrast, we did not find obvious differentiation in PLS‐DA plot with the female data; however, the Orchid Island and Mindanao PAIC clusters were significantly different from each other (Orchid Island vs. Mindanao PAIC: CER = 0.277; *p‐*value < .05; Table 1b). Nonetheless, the overall discrimination method based on PLS‐DA among the female samples was found to be nonsignificant (CER = 0.567, *p‐*value > .05; Table 1b), which is consistent with our initial PCA results.

**TABLE 1:** The results of significance test based on cross‐model validation of *A. lanyuensis* male (a) and female (b) morphological data. The significant terms in pairwise comparisons are in bold (*p*‐value < .05)

(a) Male Overall cross‐model validation test: CER = 0.512; *p‐*value = .002				
Male clusters	CER (*p* value)			
	Orchid Island	Luzon PAIC	Mindanao PAIC	Palawan PAIC
Luzon PAIC	0.21 (*p* = .0669)	–	–	
Mindanao PAIC	**0.23741 (*p* = .0225)**	**0.111 (*p* = .0038)**	–	
Palawan PAIC	0.185 (*p* = .0882)	0.563 (*p* = .6616)	0.203 (*p* = .1642)	–
Visayas PAIC	**0.191 (*p* = .0464**)	0.377 (*p* = .1556)	0.408 (*p* = .3196)	0.456 (*p* = .3587)

(b) Female Overall cross‐model validation test: CER =0.567; *p‐*value =0.126			
Female clusters	CER (*p* value)		
	Orchid Island	Luzon PAIC	Mindanao PAIC
Luzon	0.150 (0.068)	–	–
Mindanao PAIC	**0. 277 (0.038)**	0.323 (0.260)	–
Visayas PAIC	0.553 (0.721)	0.204 (0.053)	0.525 (0.708)

**FIGURE 3:**
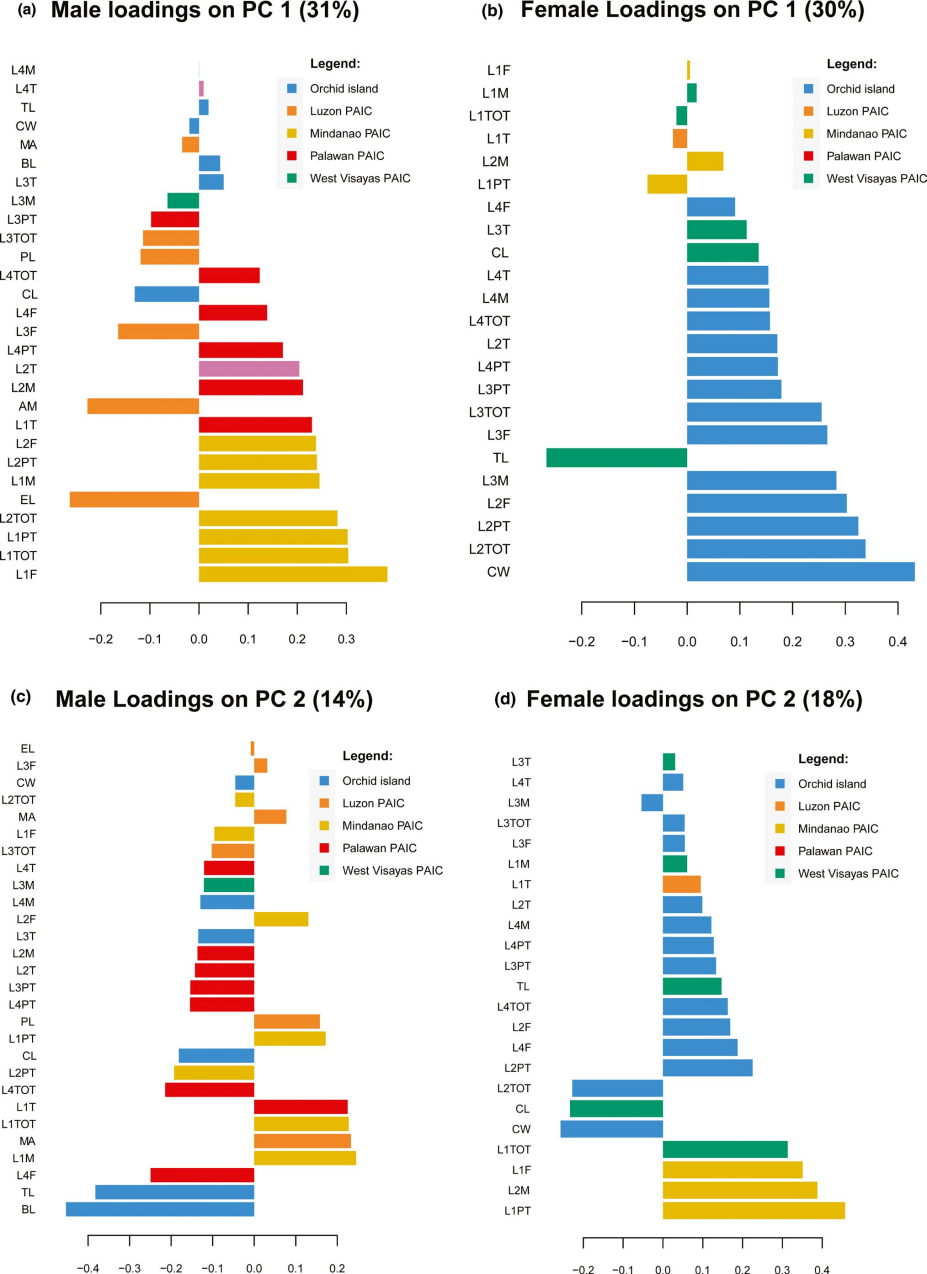
PLS‐DA loading plots for the 1st and 2nd components where colors indicate the PAICs for which the selected variable has a maximal mean value

To visualize and explore the correlations among variables, we used the latent components in the PLS‐DA to display a loading vector plot. The loading vector plot demonstrates the importance of each variable and its contribution to the overall variance in males and females. Figure 3 shows the results of the male and female loading vector plot obtained using two components from PLS‐DA. For male data, the two most important variables showed for the first component (31.00% variance explained) L1F and L1TOT, while BL and TL are the most important variables for the 2nd component (14.00% variance). These variables have substantial contributions to the variations of Mindanao samples and Orchid Island samples, respectively (Figure 3a). For females, the two most important variables using the first component (30.00% variance explained) were CW and L2TOT, while L1PT and L2M are the two most important variables for the 2nd component (18.00% variance). These variables contribute to female variation in the Orchid Island and Mindanao samples, respectively.

**FIGURE 4:**
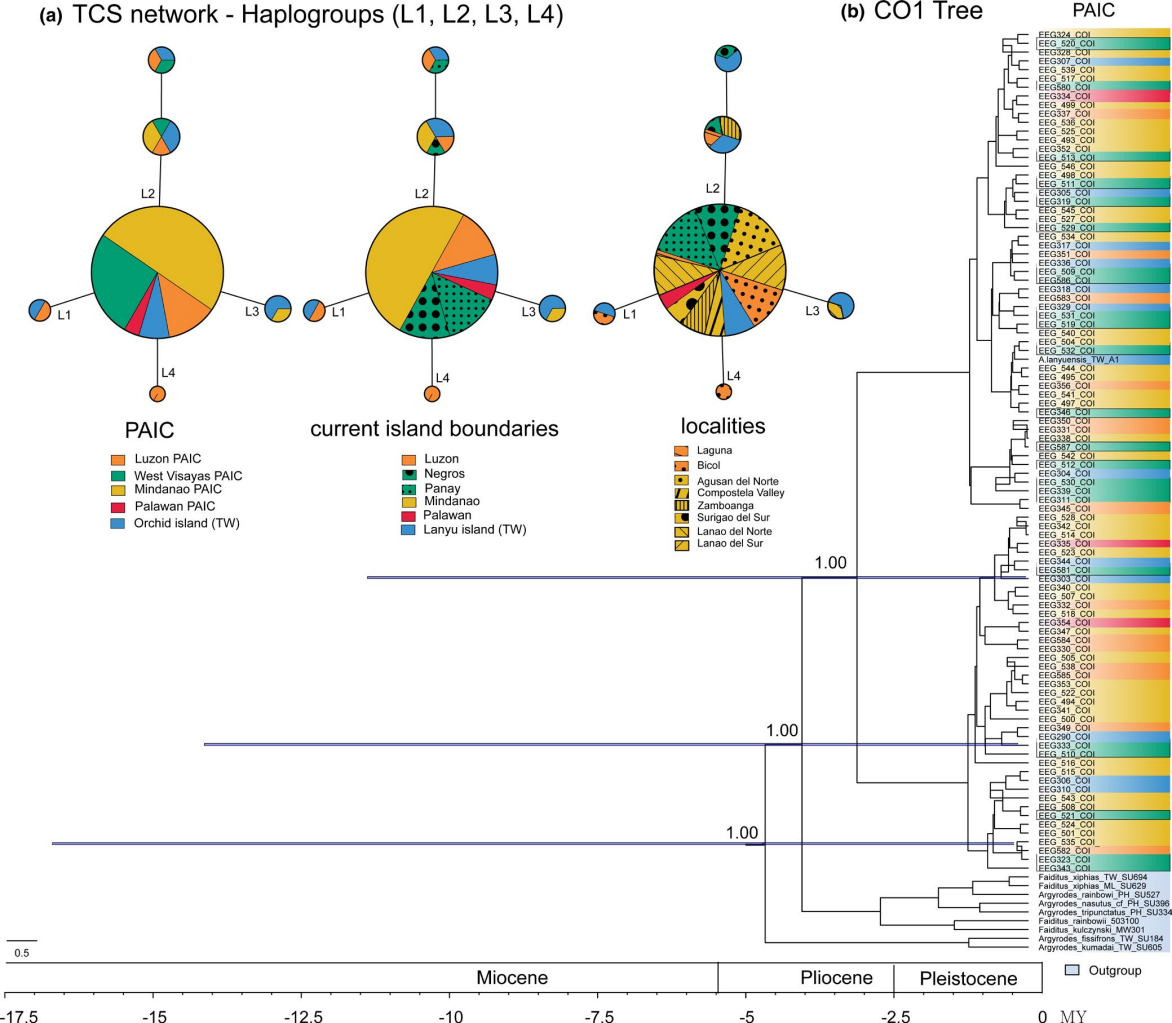
TCS network according to PAICs, current island boundaries, and localities (a); and BEAST tree using the mitochondrial CO1 gene marker (b)

We analyzed the genetic structure among all Taiwan and Philippine populations, using 840 bps of CO1 gene region. The aligned matrix showed a total nucleotide diversity (Pi) of 0.00015, and haplotype diversity (Hd) of 0.122 (Table 1). The TCS network indicated four major haplotypes (L1–L4) across our samples (Figure 4a). Geographically, the most distant population sampled is Orchid Island with four primary haplotypes. Based on haplotype diversity, Orchid Island has the highest haplotype diversity of any islands (Hd = 0.4100; Table 2). However, surprisingly, no geographic pattern in haplotype distribution can be discerned (Figure 4a). The IBD scatterplot shows a single consistent density of points suggesting a genetic homogenization (Appendix 7), which showed a weak and nonsignificant correlation between genetic and geographical distances across PAICs (*R* ^2^ = 0.02313; *p*‐value = 2.2e−16). We also obtained low pairwise *F*_ST_ values that ranged from −0.395 to 0.054 with nonsignificant *p*‐values (Appendix 8). The lack of IBD and low *F*_ST_ values suggested a limited population differentiation and high gene flow among PAIC populations and in present‐day islands.

**TABLE 2:** Haplotype and nucleotide diversities of *A. lanyuensis* collected from Taiwan and Philippines according to PAIC (a) and current geographic boundaries (b)

(a)	Orchid island	Luzon PAIC	West Visayas PAIC	Palawan PAIC	Mindanao PAIC	Total
Replicate	13	17	20	3	42	95
Haplotype	3	3	1	1	2	4
Hd	0.410	0.228	0	0	0.04762	0.122
Pi	0.00052	0.00028	0	0	0.00006	0.00015
Theta	0.00077	0.00071	0	0	0.00028	0.00070

(b)	Orchid island	Luzon island	Negros Island	Panay island	Palawan	Mindanao PAIC	Total
Replicate	13	17	10	12	3	42	95
Haplotype	3	3	1	1	1	2	4
Hd	0.410	0.228	0	0	0	0.04762	0.122
Pi	0.00052	0.00028	0	0	0	0.00006	0.00015
Theta	0.00077	0.00071	0	0	0	0.00028	0.00070

The same lack of pattern is also apparent in our BEAST maximum clade credibility (MCC) tree (Figure 4b), which shows two, strongly supported (Posterior Probability, or PP = 1.00) major clades, each of which exhibits no differentiation among PAIC or current island boundaries. Furthermore, all nodes within these two major clades have low posterior probability support (PP < 0.5), which is surprising given that CO1 is a rapidly evolving mitochondrial gene region. The divergence time of *A. lanyuensis* from the outgroup suggests that this species emerged in 3.1241 MYA (95% height posterior density: 0.2774–11.30 MYA), within the Neogene; specifically, Miocene–Pliocene epochs.

**FIGURE 5:**
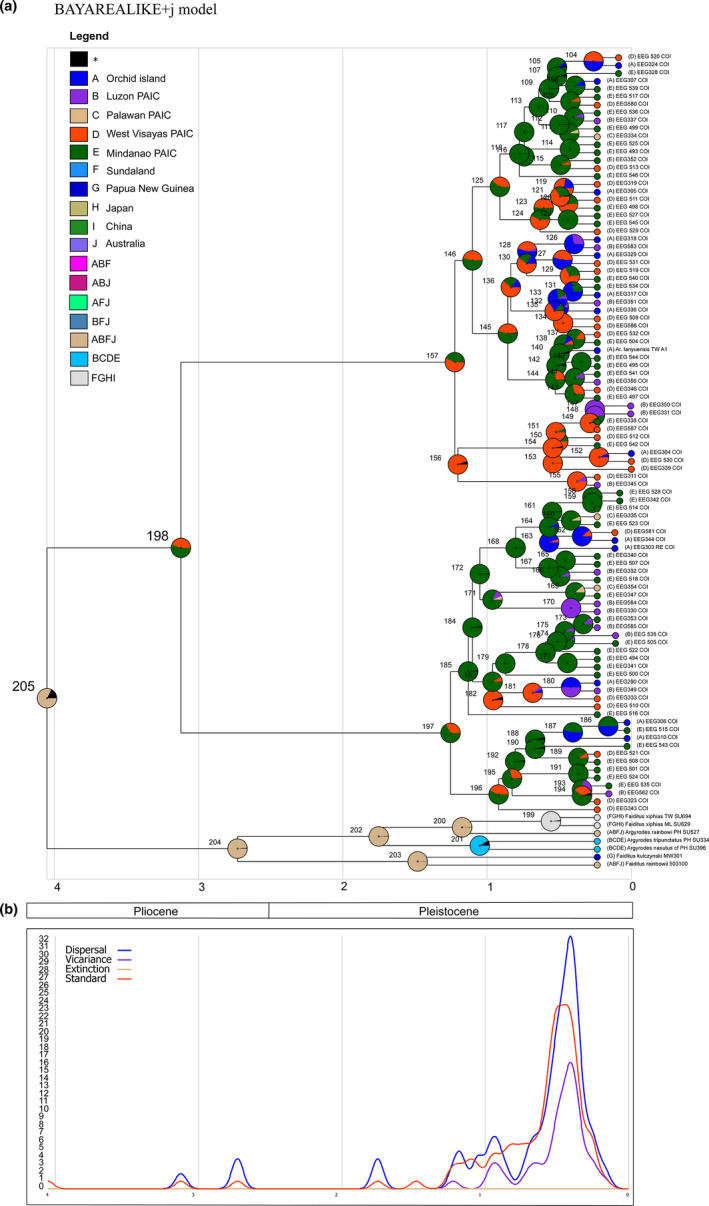
Ancestral area reconstruction from BioGeoBEARS derived from BEAST maximum clade credibility tree (a). The best‐fit model was BAYEREALIKE +J model with geologic time scale presented. Circles at each node show the most likely ancestral areas, while circles at the tips indicate the extant geographic distribution

The biogeographical analyses from the best‐fit model in BioGeoBEARS (BAYAREALIKE +j) suggested that *A. lanyuensis* most likely originated from the Mindanao PAIC [node 198; area E; marginal probability (MP) = 55.33%; Figure 5a]. A similar ancestral area was also suggested by the S‐DIVA analysis (node 198; area E; MP = 68.64%; Appendix 9), while the BBM analysis inferred both Mindanao and West Visayas PAIC as ancestral areas (node 198; area DE; MP = 48.14%; Appendix 10). Figure 5b shows the probability density of evolutionary events along the time‐calibrated tree. We observed a consistent higher probability density of dispersal events than vicariance events that started from node 198, specifically at ~3 MYA (Miocene‐Pliocene epochs) when *A. lanyuensis* diverged from the outgroup. Dispersal events continued toward later nodes wherein more dispersal events have occurred (Figure 5b). Therefore, based on our phylogenetic analyses and biogeographical reconstruction analyses, we reject the strict PAIC biogeographical patterns/predictions and the recent southward colonization (north‐to‐south prediction) and thus accept the south‐to‐north colonization as our best interpretation, but with little to no differentiation due to recent dispersal events and in response to a wide array of host species during range expansion.

## 4 DISCUSSION

Our study demonstrated an updated geographic distribution of *Argyrodes lanyuensis* that covers almost the entire Philippine Archipelago, aside from Orchid Island, Taiwan, on which this species previously was thought to be endemic (Figure 1a). This species exhibits two phenotypically differentiated units in male morphology (Orchid Island Taiwan+Luzon, Philippines populations vs. Palawan+West Visayan+Mindanao populations; Figure 2c). Our estimated divergence time suggests that this species originated *ca* 3.1241 MYA, during the Pliocene epoch (Figure 4b). Thus, it may have already existed before Pleistocene glacial fission–fusion cycles or PAIC fragmentation. We identified no genetic structure across PAIC divisions or current island boundaries based on our time‐calibrated tree and haplotype distribution (Figure 4). Additionally, the biogeographical reconstruction based on “BioGeoBEARS” and RASP suggested Mindanao as the most likely ancestral range (Figure 5; Appendix 9 and Appendix 10). Hence, our results favor south‐to‐north colonization over north‐to‐south colonization (Figure 1a) with no PAIC‐genetic‐structured variations.

The estimated divergence time of this species, which may have preceded Pleistocene glacial cycles, is inconsistent with the PAIC‐based geographically structured genetic variation. The south‐to‐north colonization appears most plausible based on our results. This species may have diverged from an ancestral lineage in Sundaland and first colonized the southern Philippine islands via the eastern island arc or/and western island arc (Figure 1a; Route 1, 2 and Figure 5). The eastern island arc follows the colonization patterns from Borneo–Sulu archipelago–Mindanao–Leyte–Samar–Luzon (Huxley, 1868), while the western island arc follows the colonization route from Borneo–Palawan–Mindoro–Luzon (Dickerson, 1928). The south‐to‐north colonization inference was also consistent based on our MCC tree with strong nodal support (PP = 1.00) obtained for the *A. lanyuensis* clade, given that the outgroups are Australasian (e.g., *A. rainbowi*, *Faiditus xiphias*; Figure 4b) and Philippine (*A. tripunctatus*) species. Similar results were obtained by Su and Smith (2014) using different genetic markers. Thus, we suggest that this species invaded from the southern Philippines, with subsequent range expansion toward northern islands, eventually including Orchid Island of Taiwan via the Batanes‐Babuyan island's route (Figure 1a; Route 3). However, further analyses of colonization patterns with higher genomic marker coverage should be explored in the Philippines, including the island chains to the south of Orchid Island to test the hypotheses of interisland, stepwise colonization (e.g., Su et al., 2016; Yang et al., 2018).

The lack of IBD (*R* ^2^ = 0.02313, *p*‐value = 2.2e−16, Appendix 7) and low *F*_ST_ values (−0.395 to 0.054; Appendix 8) imply high gene flow and limited population differentiation of *A. lanyuensis*. Based on the inference of evolutionary events using the best‐fit model in “BioGeoBEARS,” we observed a high density of recent dispersal events over vicariance (Figure 5b). These events enabled *A. lanyuensis* to disperse among islands most likely by “ballooning” with no signals of local adaptations. Even though spiders can disperse through long‐distance “ballooning,” evolutionary patterns are usually evident in these animals because of their unique ecological attributes that can be seen through their strong habitat affinities (Gillespie, 2016). For example, genetic structure was observed in excellent dispersalist, *Nephila pilipes* (Kuntner & Agnarsson, 2011; Su et al., 2007), and *Argiope bruennichi* (Krehenwinkel et al., 2016). However, we could not observe local adaptations in the case of *A. lanyuensis*. The specific behavioral phenotype of this species, which is a “generalist” kleptoparasite, could explain the limited differentiation exhibited in this species and implies higher tolerance on different host webs (a case of ecological adaptation) without specialized functions in host‐specific feeding strategies. Other spider kleptoparasites (e.g., *A. fissifrons* and *A. miniaceus*) utilize webs of specific host spiders to forage prey items (Tso & Severinghaus, 2000) and are thus called ecological “specialist” kleptoparasites (Su et al., 2018). These specialists demonstrate a strong association of these kleptoparasites to their specific host species which in turn may have caused genetic‐structured populations across different islands in the Australasian region (Su & Smith, 2014). Thus, we assume that specialized kleptoparasitism could interrupt gene flow between different groups or geographic populations and might promote speciation, in contrast to generalist kleptoparasites (e.g., *A. lanyuensis*). The pilot study on terrestrial invertebrates, the Philippine endemic treehopper, *Pyrgonota bifoliata* (Membracidae), that applies a similar PAIC model of speciation shows more evident population subdivisions among PAIC islands (Su et al., 2014). Each subpopulation of *P. bifoliate* appears to specialize on a species‐specific host plant, per PAIC island (Su et al., 2014). In contrast, the results presented here could be a special case for the Philippines archipelago in that we estimate a deeper, pre‐Pleistocene temporal divergence time, and yet we did not detect any clear differentiation among PAICs or modern, current‐day islands.

The phenotypic clustering evident in males from Orchid Island (southern Taiwan) and Luzon Island (northern Philippines) may suggest founder effects or could be related to sexual selection. The possible colonization of *A. lanyuensis* from the southern portions of the archipelago toward northern islands and eventually Orchid Island might have led to founder events. The most important variables contributing to clustering patterns of males are lengths of the first legs (L1F, L1TOT; Figure 3a) and palp bulb length (BL; Figure 3b). These variables contribute greatly to the samples from the inferred ancestral range (Mindanao island) and the recent population from Orchid Island, respectively. The phenotypic variations observed in these two populations, specifically in the leg I and palp bulb, could be attributed to sexual selection in males. Male A. lanyuensis typically have longer Leg I than females (Yoshida et al., 1998), in which similar observations were recorded in this study (Appendix 2). Leg 1 was usually used by both male and female *A. lanyuensis*, for moving around the web to locate the host's silk and prey items for food consumption (Yoshida et al., 1998). For most of the *Argyrodes* spiders, leg I is very important in the male–male competition for female copulation, which results from highly modified intrasexual selection in males (Whitehouse, 1991, 2016). With these phenotypic variations in the leg I and palp bulb lengths between the ancestral (Mindanao) and recent populations (Orchid Island), we hypothesize that different mating strategies may evolve in recent populations given the selective pressures in the new environment. In argyrodinae spiders, species‐specific differences and intersexual differences in foraging strategies have been noted (Cangialosi, 1990; Kerr, 2005; Tso & Severinghaus, 2000). Female *A. lanyuensis* may be able to utilize the same foraging strategies across different spider hosts in which the functional genes for a specialized foraging behavior are not well expressed, even though a unique form of foraging strategy (host silk consumption) has been noted on this species in Orchid Island (Yoshida et al., 1998). Hence, our results on the population structure of females could be related to their foraging behavior. On the other hand, mating strategies in males could lead to the morphological differentiation of this spider kleptoparasite generalist (Whitehouse, 2016). However, these results should be further validated due to the limited sample size and genetic markers.

Our study demonstrates the possible exchange of taxa between two geographical entities. In this case, faunal transfers (dispersal) were possible between Taiwan and the Philippines through the Luzon‐Taiwan strait, in which dispersal events originated from the Philippines. This study added to the cases of Philippine fauna that have been recorded to disperse from the Philippines to Orchid Island. These include *Eutropis cumingi* (skink; Ota & Huang, 2000), *Polypedates leucomystax* (frog; Kuraishi et al., 2009; Ota, 2004); five species of geckos (Ota, 1987; Siler et al., 2014; Wang, 1962), two species of butterflies, *Macroglossum ungues cheni* (Yen et al., 2003), and *Catopyrops ancyra almora* (Lu & Hsu, 2002). In contrast, other taxa (e.g., plants, shrews) have been recorded to disperse in the Philippines from Orchid Island (south‐to‐north colonization; Dickerson, 1928; Esselstyn & Oliveros, 2010; Oliveros & Moyle, 2010). Thus, careful analyses should be done for the diversification of taxa along the Philippine‐Lanyu oceanic island chain.

## 5 CONCLUSION

In conclusion, our results revealed the presence and widespread distribution of *A. lanyuensis* in the Philippines, far beyond its originally assumed microendemic distribution in Orchid Island, Taiwan. Our study also emphasized northward colonization of *A. lanyuensis* from the Philippines toward adjacent Orchid Island, Taiwan, through recent dispersal events. The molecular data highlight the importance of behavioral phenotype such as foraging behavior, rather than isolation by distance, sea‐level vicariance, and climatic oscillations (e.g., PAICs Paradigm biogeographic isolation) as drivers of diversification of kleptoparasitic spiders. However, it is also important to note the structured variations we observed in males for the northern populations, which possibly attributed to mating strategies. Future work on this study system may be best informed by a higher coverage of genomic data, to get a more robust and finely differentiated characterization of its population structure across the Philippines, northwards directing to Orchid Island.

## CONFLICT OF INTERESTS

The authors declare no competing interests.

## AUTHOR CONTRIBUTIONS

**Mae Responte:** Formal analysis (lead); Investigation (lead); Methodology (equal); Software (lead); Visualization (lead); Writing‐original draft (lead). **Yi‐Fan Chiu:** Formal analysis (supporting); Methodology (equal); Software (supporting). **Po Peng:** Formal analysis (supporting); Methodology (equal); Software (supporting); Visualization (supporting). **Rafe M. Brown:** Methodology (equal); Validation (equal); Writing‐review & editing (equal). **Chia‐Yen Dai:** Supervision (equal); Validation (equal); Writing‐review & editing (equal). **Yong‐Chao Su:** Conceptualization (lead); Funding acquisition (lead); Methodology (equal); Project administration (equal); Supervision (lead); Validation (equal); Writing‐review & editing (lead).

## Data Availability

DNA sequences: GenBank accessions MN881069‐ MN881072; KJ648441.1; KJ648369.1; KJ648430.1; KJ648426.1; KJ648436.1; MW549752; MW549751; KJ648385.1. Responte et al. (2021), Dryad, Dataset, https://doi.org/10.5061/dryad.1ns1rn8tk.

## APPENDIX 1

(a) Collection information of *Argyrodes lanyuensis* samples used for morphological and molecular analyses with GenBank accession number

**Table:**

No.	Sample number	PAIC	Island boundary	Latitude/Longitude	Gender (M/F/subadult)	Molecular Analyses (DNA No.)	GenBank accession number	Morphological Analyses (Yes/No)
1	SU56.57.58–1	Luzon	Luzon	13.663188/123.3325	F	EEG 582	MN881070	Yes
2	SU56.57.58–2	Luzon	Luzon	13.663188/123.3325	M	EEG 583	MN881070	Yes
3	SU56.57.58–3	Luzon	Luzon	13.663188/123.3325	F	EEG 584	MN881070	No
4	Su54.55A	Luzon	Luzon	13.663188/123.3325	Subadult	EEG 356	MN881070	No
5	SU54.55B	Luzon	Luzon	13.663188/123.3325	F	EEG 585	MN881070	Yes
6	Su40.41A	Luzon	Luzon	13.663188/123.3325	M	EEG 349	MN881071	Yes
7	SU40.41B	Luzon	Luzon	13.663188/123.3325	F	EEG 350	MN881070	Yes
8	SU40–41C	Luzon	Luzon	13.663188/123.3325	Subadult	EEG 519	MN881070	No
9	SU40–41D	Luzon	Luzon	13.663188/123.3325	Subadult	EEG 520	MN881070	No
10	SU42–43	Luzon	Luzon	13.663188/123.3325	Subadult	EEG 521	MN881070	No
11	SU46.47B	Luzon	Luzon	13.663188/123.3325	M	EEG 351	MN881070	Yes
12	Su52.53A	Luzon	Luzon	13.663188/123.3325	M	EEG 330	MN881070	No
13	Su52.53B	Luzon	Luzon	13.663188/123.3325	F	EEG 331	MN881070	No
14	Su52.53C	Luzon	Luzon	13.663188/123.3325	F	EEG 332	MN881070	No
15	Su32.33	Luzon	Luzon	14.121641/121.335872	Subadult	EEG 345	MN881070	No
16	Su34.35A	Luzon	Luzon	14.121641/121.335872	Subadult	EEG 337	MN881070	No
17	SU60 61	Luzon	Luzon	13.663188/123.3325	M	EEG 538	MN881072	No
18	Su355B	Palawan	Palawan	9.56277778/126.228372	Subadult	EEG 335	MN881070	No
19	Su355A	Palawan	Palawan	9.56277778/126.228372	Subadult	EEG 334	MN881070	No
20	Su347A	Palawan	Palawan	9.56277778/126.228372	Subadult	EEG 354	MN881070	No
21	SU141–142–143 A	West Visayas	Panay	11.026671/122.658891	F	EEG 529	MN881070	Yes
22	SU141–142–143 B	West Visayas	Panay	11.026671/122.658891	F	EEG 530	MN881070	Yes
23	SU141–142–143 C	West Visayas	Panay	11.026671/122.658891	M	EEG 531	MN881070	Yes
24	SU141–142–143 D	West Visayas	Panay	11.026671/122.658891	M	EEG 532	MN881070	Yes
25	SU147–148A	West Visayas	Panay	11.026671/122.658891	Subadult	EEG 511	MN881070	No
26	SU147–148 B	West Visayas	Panay	11.026671/122.658891	Subadult	EEG 512	MN881070	No
27	SU107	West Visayas	Negros	10.50888889/123.1052778	F	EEG 319	MN881070	No
28	SU110.111–A	West Visayas	Negros	10.50888889/123.1052778	F	EEG 586	MN881070	Yes
29	SU110.111–B	West Visayas	Negros	10.50888889/123.1052778	F	EEG 587	MN881070	Yes
30	SU110–111‐C	West Visayas	Negros	10.50888889/123.1052778	Subadult	EEG 510	MN881070	No
31	SU134–A	West Visayas	Panay	11.026671/122.658891	F	EEG 580	MN881070	Yes
32	SU 134–B	West Visayas	Panay	11.026671/122.658891	Subadult	EEG 581	MN881070	No
33	Su118	West Visayas	Negros	10.50888889/123.1052778	F	EEG 333	MN881070	No
34	Su114.115.116B	West Visayas	Negros	10.50888889/123.1052778	Subadult	EEG 346	MN881070	No
35	Su106–2	West Visayas	Negros	10.50888889/123.1052778	F	EEG 311	MN881070	Yes
36	Su94.95.96	West Visayas	Negros	10.50888889/123.1052778	Subadult	EEG 339	MN881070	No
37	SU97–98	West Visayas	Negros	10.50888889/123.1052778	F	EEG 509	MN881070	Yes
38	SU145–146	West Visayas	Panay	11.026671/122.658891	M	EEG 513	MN881070	Yes
39	Su138.139B	West Visayas	Panay	11.026671/122.658891	M	EEG 323	MN881070	Yes
40	Su108.109	West Visayas	Negros	10.50888889/123.1052778	Subadult	EEG 343	MN881070	No
41	SU2096	Mindanao	Mindanao	9.052554/125.610306	Subadult	EEG 545	MN881070	No
42	SU2106	Mindanao	Mindanao	7.891919/123.77847	Subadult	EEG 525	MN881070	No
43	Su320.321A	Mindanao	Mindanao	7.58555556/125.9865278	F	EEG 340	MN881070	Yes
44	Su320.321A	Mindanao	Mindanao	7.58555556/125.9865278	Subadult	EEG 347	MN881070	No
45	SU320–321 B	Mindanao	Mindanao	7.58555556/125.9865278	Subadult	EEG 527	MN881070	No
46	SU320–321 C	Mindanao	Mindanao	7.58555556/125.9865278	Subadult	EEG 528	MN881070	No
47	SU2102	Mindanao	Mindanao	9.052554/125.610306	F	EEG 499	MN881070	Yes
48	SU2103A	Mindanao	Mindanao	9.052554/125.610306	Subadult	EEG 500	MN881070	No
49	SU2103B	Mindanao	Mindanao	9.052554/125.610306	Subadult	EEG 501	MN881070	No
50	SU2126C	Mindanao	Mindanao	7.637164/124.045471	Subadult	EEG 504	MN881070	No
51	Su318.319A	Mindanao	Mindanao	7.58555556/125.9865278	Subadult	EEG 342	MN881070	No
52	SU318–319B	Mindanao	Mindanao	7.58555556/125.9865278	Subadult	EEG 523	MN881070	No
53	SU2089	Mindanao	Mindanao	9.052554/125.610306	F	EEG 524	MN881070	Yes
54	SU118B	Mindanao	Mindanao	7.637164/124.045471	F	EEG 507	MN881070	No
55	SU118C	Mindanao	Mindanao	7.637164/124.045471	M	EEG 508	MN881070	No
56	SU2076A	Mindanao	Mindanao	8.172717/126.228372	F	EEG 493	MN881070	Yes
57	SU2076B	Mindanao	Mindanao	8.172717/126.228372	F	EEG494	MN881070	Yes
58	SU2086A	Mindanao	Mindanao	8.172717/126.228372	Subadult	EEG 495	MN881070	No
59	SU2086 C	Mindanao	Mindanao	8.172717/126.228372	F	EEG 497	MN881070	No
60	SU2109 B	Mindanao	Mindanao	7.891919/123.77847	M	EEG 534	MN881070	Yes
61	SU2109 C	Mindanao	Mindanao	7.891919/123.77847	Subadult	EEG 535	MN881070	No
62	SU2097	Mindanao	Mindanao	9.052554/125.610306	Subadult	EEG 541	MN881070	No
63	SU2095	Mindanao	Mindanao	9.052554/125.610306	F	EEG 542	MN881070	Yes
64	SU2098	Mindanao	Mindanao	9.052554/125.610306	Subadult	EEG 544	MN881070	No
65	SU2101	Mindanao	Mindanao	9.052554/125.610306	Subadult	EEG 498	MN881070	No
66	Su408A	Mindanao	Mindanao	7.0075/122.0230556	Subadult	EEG 352	MN881070	No
67	Su408B	Mindanao	Mindanao	7.0075/122.0230556	Subadult	EEG 353	MN881070	No
68	SU408C	Mindanao	Mindanao	7.0075/122.0230556	Subadult	EEG 540	MN881070	No
69	SU408 D	Mindanao	Mindanao	7.0075/122.0230556	F	EEG 539	MN881070	No
70	SU2110A	Mindanao	Mindanao	7.891919/123.77847	F	EEG 515	MN881069	Yes
71	SU2110B	Mindanao	Mindanao	7.891919/123.77847	F	EEG 516	MN881070	Yes
72	SU2110C	Mindanao	Mindanao	7.891919/123.77847	M	EEG 517	MN881070	Yes
73	SU2099	Mindanao	Mindanao	9.052554/125.610306	F	EEG 543	MN881070	No
74	SU367	Mindanao	Mindanao	7.0075/122.0230556	Subadult	EEG 328	MN881070	No
75	Su405A	Mindanao	Mindanao	7.0075/122.0230556	M	EEG 338	MN881070	No
76	SU405B	Mindanao	Mindanao	7.0075/122.0230556	F	EEG 536	MN881070	No
77	SU2091	Mindanao	Mindanao	9.052554/125.610306	Subadult	EEG 546	MN881070	No
78	Su400A	Mindanao	Mindanao	7.0075/122.0230556	F	EEG 341	MN881070	Yes
79	SU2107	Mindanao	Mindanao	7.891919/123.77847	M	EEG 514	MN881070	Yes
80	SU2126D	Mindanao	Mindanao	7.637164/124.045471	Subadult	EEG 505	MN881070	No
81	SU2108	Mindanao	Mindanao	7.891919/123.77847	F	EEG 518	MN881070	Yes
82	SU314–315	Mindanao	Mindanao	7.58555556/125.9865278	F	EEG 522	MN881070	Yes
83	SU511	–	Orchid island	22.00972222/121.570865	Subadult	EEG 306	MN881069	No
84	SU489	–	Orchid island	22.00972222/121.570865	Subadult	EEG 310	MN881069	No
85	Su114A	–	Orchid island	22.00972222/121.570865	Subadult	EEG 336	MN881070	No
86	Su503	–	Orchid island	22.00972222/121.570865	Subadult	EEG 329	MN881070	No
87	Su181.182.M1	–	Orchid island	22.00972222/121.570865	M	EEG 305	MN881070	Yes
88	Su284.285	–	Orchid island	22.00972222/121.570865	Subadult	EEG 317	MN881070	No
89	Su520	–	Orchid island	22.00972222/121.570865	Subadult	EEG 304	MN881070	No
90	Su483	–	Orchid island	22.00972222/121.570865	Subadult	EEG 307	MN881070	No
91	Su508	–	Orchid island	22.00972222/121.570865	Subadult	EEG 324	MN881070	No
92	Su521	–	Orchid island	22.00972222/121.570865	Subadult	EEG 344	MN881070	No
93	Su177.178	–	Orchid island	22.00972222/121.570865	F	EEG 303	MN881070	Yes
94	Su499	–	Orchid island	22.00972222/121.570865	Subadult	EEG 318	MN881070	No
95	SU175	–	Orchid island	22.00972222/121.570865	F	EEG 290	MN881071	Yes
96	SU46.47A	Luzon	Luzon	13.663188/123.3325	M	–	–	Yes
97	SU46.47C	Luzon	Luzon	13.663188/123.3325	M	–	–	Yes
98	SU46.47D	Luzon	Luzon	13.663188/123.3325	F	–	–	Yes
99	SU138_139 M	West Visayas	Panay	11.026671/122.658891	M	–	–	Yes
100	SU141_142_143E	West Visayas	Panay	11.026671/122.658891	M	–	–	Yes
101	SU138_139F	West Visayas	Panay	11.026671/122.658891	F	–	–	Yes
102	SU134_135A	West Visayas	Panay	11.026671/122.658891	F	–	–	Yes
103	SU2115	Mindanao	Mindanao	7.637164/124.045471	M	–	–	Yes
104	SU2116	Mindanao	Mindanao	7.637164/124.045471	M	–	–	Yes
105	SU2117M1	Mindanao	Mindanao	7.637164/124.045471	M	–	–	Yes
106	SU2117M2	Mindanao	Mindanao	7.637164/124.045471	M	–	–	Yes
107	SU2118	Mindanao	Mindanao	7.637164/124.045471	M	–	–	Yes
108	SU2090	Mindanao	Mindanao	9.052554/125.610306	M	–	–	Yes
109	SU325M1	Mindanao	Mindanao	8.056852/126.219838	M	–	–	Yes
110	SU325M2	Mindanao	Mindanao	8.056852/126.219838	M	–	–	Yes
111	SU325M3	Mindanao	Mindanao	8.056852/126.219838	M	–	–	Yes
112	SU2111	Mindanao	Mindanao	7.891919/123.77847	F	–	–	Yes
113	SU2114	Mindanao	Mindanao	7.637164/124.045471	F	–	–	Yes
114	SU2117	Mindanao	Mindanao	7.637164/124.045471	F	–	–	Yes
115	SU2118F1	Mindanao	Mindanao	7.637164/124.045471	F	–	–	Yes
116	SU2118F2	Mindanao	Mindanao	7.637164/124.045471	F	–	–	Yes
117	SU2092	Mindanao	Mindanao	9.052554/125.610306	F	–	–	Yes
118	SU2102F2	Mindanao	Mindanao	9.052554/125.610306	F	–	–	Yes
119	SU407	Mindanao	Mindanao	7.0075/122.0230556	F	–	–	Yes
120	SU181_182M2	–	Orchid island	22.0096111/121.57086111111111	M	–	–	Yes
121	SU484	–	Orchid island	22.0096111/121.57086111111111	M	–	–	Yes
122	SU487	–	Orchid island	22.0096111/121.57086111111111	M	–	–	Yes
123	SU491	–	Orchid island	22.0096111/121.57086111111111	M	–	–	Yes
124	SU1351	–	Orchid island	22.0295240264385/121.57560297288	M	–	–	Yes
125	SU1368	–	Orchid island	22.0092420000582/121.572734015062	M	–	–	Yes
126	SU1387	–	Orchid island	22.0097780227661/121.574569987133	M	–	–	Yes
127	SU1697	–	Orchid island	22.6631840039044/121.501822024583	M	–	–	Yes
128	SU1706M	–	Orchid island	22.0291449967771/121.576519031077	M	–	–	Yes
129	SU181_182F1	–	Orchid island	22.0096111/121.57086111111111	F	–	–	Yes
130	SU181_182F2	–	Orchid island	22.0096111/121.57086111111111	F	–	–	Yes
131	SU484	–	Orchid island	22.0096111/121.57086111111111	F	–	–	Yes
132	SU1383	–	Orchid island	22.0095020066946/121.573526021093	F	–	–	Yes
133	SU1701	–	Orchid island	22.0281270146369/121.577498959377	F	–	–	Yes
134	SU1706M	–	Orchid island	22.0291449967771/121.576519031077	F	–	–	Yes

(b) Collection information of outgroup samples used for molecular analyses with GenBank accession number

**Table:**

Species	Sample number	Accession number
*Faiditus xiphias*	SU694	KJ648441.1
*Faiditus xiphias*	SU629	KJ648369.1
*Argyrodes rainbowi*	SU527	KJ648430.1
*Argyrodes nasutus*	SU396	KJ648426.1
*Argyrodes tripunctatus*	SU334	KJ648436.1
*Arygyrodes rainbowi*	503100	MW549752
*Argyrodes kulczynski*	MW301	MW549751
*Argyrodes fissifrons*	SU184	KJ648385.1
*Argyrodes kumadai*	SU605	KJ648387.1

## APPENDIX 2

Total number of individuals examined per PAIC island

**Table:**

PAIC	Number of individuals examined for morphological analyses		Number of individuals examined for molecular analyses
	Male	Female	
Orchid island	10	8	13
Luzon	5	4	13
Palawan	3	–	3
West Visayas	6	8	23
Mindanao	13	18	43
Total number of Individuals	37	38	95

Abbreviation: PAIC, Pleistocene Aggregate Island Complexes.

## APPENDIX 3

Morphometrics of *A. lanyuensis* male and female samples with 28 and 23 variables, respectively

**Table:**

Characters code	Characters definition	Male (*N* = 37)		Female (*N* = 38)	
		Mean ± *SD* (mm)	Range (mm)	Mean ± *SD* (mm)	Range (mm)
CL	Carapace Length	1.396 ± 0.147	1.12 ± 1.804	1.158 ± 0.088	0.918 ± 1.326
TL	Total Length	2.198 ± 0.145	1.900 ± 2.631	0.644 ± 0.630	0.352 ± 2.947
CW	Carapace Width	0.453 ± 0.073	0.344 ± 0.604	2.440 ± 0.589	0.645 ± 3.173
L1F	Leg I Femur	2.474 ± 0.486	1.010 ± 3.297	2.621 ± 0.227	1.998 ± 3.128
L1PT	Leg I Patella & Tibia	2.439 ± 0.243	1.945 ± 3.112	2.403 ± 0.239	1.738 ± 2.802
L1M	Leg I Metatarsus	2.294 ± 0.342	0.831 ± 3.068	2.231 ± 0.298	0.788 ± 2.683
L1T	Leg I Tarsus	0.930 ± 0.097	0.689 ± 1.108	0.935 ± 0.096	0.630 ± 1.119
L1TOT	Leg I Total Length	8.239 ± 0.875	6.368 ± 10.585	8.190 ± 0.690	6.696 ± 9.648
L2F	Leg II Femur	1.033 ± 0.159	0.303 ± 1.227	1.025 ± 0.108	0.815 ± 1.257
L2PT	Leg II Patella &Tibia	0.922 ± 0.100	0.623 ± 1.099	0.845 ± 0.106	0.578 ± 1.033
L2M	Leg 2 Metatarsus	0.696 ± 0.071	0.445 ± 0.807	0.725 ± 0.102	0.439 ± 0.929
L2T	Leg II Tarsus	0.413 ± 0.058	0.314 ± 0.493	0.426 ± 0.056	0.303 ± 0.509
L2TOT	Leg II Total Length	3.064 ± 0.301	2.237 ± 3.508	0.678 ± 0.923	0.231 ± 3.569
L3F	Leg III Femur	0.550 ± 0.074	0.416 ± 0.770	0.555 ± 0.101	0.388 ± 0.921
L3PT	Leg III Patella&Tibia	0.413 ± 0.058	0.295 ± 0.626	0.428 ± 0.076	0.323 ± 0.686
L3M	Leg III Metatarsus	0.306 ± 0.036	0.234 ± 0.418	0.319 ± 0.064	0.207 ± 0.532
L3T	Leg III Tarsus	0.243 ± 0.042	0.156 ± 0.339	0.268 ± 0.041	0.187 ± 0.347
L3TOT	Leg III Total Length	1.512 ± 0.167	1.196 ± 2.153	1.569 ± 0.247	1.224 ± 2.484
L4F	Leg IV Femur	0.879 ± 0.094	0.603 ± 1.010	0.879 ± 0.183	0.401 ± 1.153
L4PT	Leg IV Patella &Tibia	0.648 ± 0.083	0.413 ± 0.852	0.636 ± 0.129	0.324 ± 0.867
L4M	Leg IV Metatarsus	0.467 ± 0.055	0.311 ± 0.654	0.481 ± 0.094	0.205 ± 0.693
L4T	Leg IV Tarsus	0.309 ± 0.036	0.223 ± 0.380	0.324 ± 0.064	0.174 ± 0.512
L4TOT	Leg IV Total Length	2.303 ± 0.223	1.582 ± 2.811	2.320 ± 0.405	1.186 ± 3.225
PL	Palpal Length	2.288 ± 0.208	1.983 ± 2.749	–	–
BL	Bulb length	0.547 ± 0.049	0.414 ± 0.627	–	–
MA	Median Apophysis	0.164 ± 0.024	0.112 ± 0.238	–	–
AP	Accessorial Apophysis	0.167 ± 0.034	0.093 ± 0.264	–	–
EL	Embolus length	0.834 ± 0.091	0.557 ± 1.053	–	–

## APPENDIX 4

PLS‐DA loading weight values for the 28 variables of males and 23 variables for females listed from the most important to the least important variable

**Table:**

Male (*n* = 37)				Female (*n* = 38)			
Characters*	Component 1	Characters	Component 2	Characters*	Component 1	Characters	Component 2
L1F	0.38351198	BL	−0.45315538	CW	0.43181394	L1PT	0.45755393
L1TOT	0.30376023	TL	−0.38248365	L2TOT	0.33830543	L2M	0.38784592
L1PT	0.3027413	L4F	−0.24934683	L2PT	0.32477021	L1F	0.35094616
L2TOT	0.28166924	L1M	0.24539086	L2F.	0.30267685	L1TOT	0.31340983
EL	−0.263136	MA	0.23294068	L3M	0.28311415	CW	−0.25730231
L1M	0.24531684	L1TOT	0.22777035	TL	−0.26712752	CL	−0.23350157
L2PT	0.23990613	L1T	0.22514304	L3F	0.26598773	L2TOT	−0.22787851
L2F	0.23806233	L4TOT	−0.21431921	L3TOT	0.25500646	L2PT	0.22516641
L1T	0.22988256	L2PT	−0.1927407	L3PT	0.17869766	L4F	0.18745189
AP	−0.22721389	CL	−0.18158622	L4PT	0.17201315	L2F	0.16876584
L2M	0.21167709	L1PT	0.17199691	L2T	0.17095882	L4TOT	0.16272747
L2T	0.20418632	PL	0.15818057	L4TOT	0.15700943	TL	0.14710868
L4PT	0.17081701	L4PT	−0.15451271	L4M	0.15582143	L3PT	0.1333082
L3F	−0.16459783	L3PT	−0.15382933	L4T	0.15414956	L4PT	0.12769284
L4F	0.13855817	L2T	−0.14256483	CL	0.1355591	L4M	0.1216094
CL	−0.13085052	L2M	−0.13641849	L3T	0.11292653	L2T	0.09883316
L4TOT	0.12354748	L3T	−0.13456348	L4F	0.09088741	L1T	0.09503677
PL	−0.11906856	L2F	0.13064304	L1PT	−0.07524103	L1M	0.06081118
L3TOT	−0.11391175	L4M	−0.1295035	L2M	0.06875709	L3F	0.05480712
L3PT	−0.09760581	L3M	−0.12116636	L1T	−0.02694092	L3TOT	0.05449961
L3M	−0.06407602	L4T	−0.12047511	L1TOT	−0.02037519	L3M	−0.05382282
L3T	0.05026468	L3TOT	−0.10240951	L1M	0.01787341	L4T	0.05072841
BL	0.04300805	L1F	−0.09576307	L1F.S	0.00526043	L3T	0.03043119
MA	−0.0342114	AP	0.0774619	–	–	–	–
CW	−0.01969183	L2TOT	−0.04569395	–	–	–	–
TL	0.01933653	CW	−0.04537034	–	–	–	–
L4T	0.00938999	L3F	0.0314079	–	–	–	–
L4M	0.00025733	EL	−0.00843207	–	–	–	–

## APPENDIX 5

Body and male palp characters used for multivariate analyses (Please refer to Appendix 3 for the definition of each character.)

## APPENDIX 6

PLS‐DA score plots of *A. lanyuensis* males (a–b) and females (c–d) based on 28 and 265 morphometrics, respectively. Individuals are plotted against components 1 and 2, grouped according to current island boundary (a and c) and locality (b and d)

## APPENDIX 7

Isolation by distance (IBD) analysis using mantel test between the Dgeo (spatial Euclidean distance) and Dgen (Edward's genetic distance). Color contours indicate kernel density estimation, where higher densities are shown by red color

## APPENDIX 8

Pairwise *F*_ST_ estimates among island boundaries. Red colored cells show *F*_ST_ values, while blue colored cells show the corresponding *p*‐values

**Table:**

Current island boundary	Orchid	Panay	Mindanao	Negros	Luzon	Palawan
Orchid	NA	−0.030	0.054	−0.075	−0.080	−0.339
Panay	0.922	NA	0.000	0.000	−0.093	0.000
Mindanao	0.000	0.901	NA	−0.124	0.001	−0.355
Negros	0.998	0.000	0.901	NA	−0.132	0.000
Luzon	0.994	0.998	0.294	0.998	NA	−0.395
Palawan	0.998	0.000	0.901	0.000	0.998	NA

## APPENDIX 9

Statistical dispersal‐vicariance S‐DIVA biogeographical reconstruction analysis using RASP

## APPENDIX 10

Bayesian binary MCMC biogeographical reconstruction analysis using RASP
